# Treatment of Rupture of the Kidney

**Published:** 1897-06

**Authors:** 


### TREATMENT OF RUPTURE OF THE KIDNEY.

Keen (*Annals of Surgery*, August, 1896), in concluding an elaborate paper on the Treatment of Traumatic Lesions of the Kidney, based on tables of 155 cases, discusses the indications for operative intervention in cases of subcutaneous rupture of this organ. Of 118 cases of this injury that have been published since 1878, fifty were fatal. On excluding twelve cases of associated injuries of other organs, two cases in which death occurred very soon after the injury, one case in which the patient possessed a single kidney, and an uncertain case, thirty-four cases are left, in fourteen of which the fatal result was due to primary, continuous, and secondary hemorrhage combined with shock, while suppuration, including peritonitis, destroyed sixteen. In four cases only was death caused by coma, anuria, and nephritis. These figures support the view held by the author, that the dangers of rupture of the kidney are especially hemorrhage and sepsis. A more frequent resort to primary nephrectomy would, it is held, have avoided a number of deaths from both of those causes. The duty of the surgeon, it is pointed out, seems clear. Where the symptoms are threatening, particularly if there be decided evidence of hemorrhage, or probable danger of sepsis, an exploratory operation should be performed without delay. The great mass of recoveries in rupture of the kidney are the slighter cases; the graver cases do not recover unless an operation is done. In any case, therefore, with severe or dangerous symptoms, the surgeon should lean toward exploration, and in severe laceration toward early nephrectomy. Hematuria is regarded as being valuable only as a symptom showing the fact of rupture of the kidney, but not as a symptom by which to decide on operating. Not the visible loss of blood by the bladder, but the easily overlooked, but far more dangerous bleeding into the perinephric tissues, or into the peritoneal cavity, should receive the chief attention. If, then, a tumor form quickly in the lumbar region, an exploratory operation in the loin should be immediately made, and if the kidney be found hopelessly destroyed, or the hemorrhage such as to require ligation of the renal vessels, nephrectomy should be practiced.—*British Medical Journal.*

